# Usefulness of Size-Exclusion Chromatography–Multi-Angle Light Scattering to Assess Particle Composition and Protein Impurities for Quality Control of Therapeutic Exosome Preparations

**DOI:** 10.3390/pharmaceutics16121526

**Published:** 2024-11-27

**Authors:** Hirotaka Nishimura, Noritaka Hashii, Tomofumi Yamamoto, Yuchen Sun, Takumi Miura, Yoji Sato, Akiko Ishii-Watabe

**Affiliations:** 1 Division of Biological Chemistry and Biologicals, National Institute of Health Sciences, 3-25-26 Tonomachi, Kawasaki-ku, Kawasaki 210-9501, Kanagawa, Japan; h_nishimura@nihs.go.jp (H.N.);; 2 Division of Medicinal Safety Science, National Institute of Health Sciences, 3-25-26 Tonomachi, Kawasaki-ku, Kawasaki 210-9501, Kanagawa, Japan; 3 Division of Cell-Based Therapeutic Products, National Institute of Health Sciences, 3-25-26 Tonomachi, Kawasaki-ku, Kawasaki 210-9501, Kanagawa, Japan; 4 Division of Drugs, National Institute of Health Sciences, 3-25-26 Tonomachi, Kawasaki-ku, Kawasaki 210-9501, Kanagawa, Japan

**Keywords:** extracellular vesicles, size exclusion chromatography, multi-angle light scattering, EV therapeutics, quality control

## Abstract

**Background:** Extracellular vesicles (EVs), including exosomes, are promising pharmaceutical modalities. They are purified from cell culture supernatant; however, the preparation may contain EVs with the desired therapeutic effects and different types of EVs, lipoproteins, and soluble proteins. Evaluating the composition of particulate impurities and the levels of protein impurities in final preparations is critical for quality control. However, few analytical methods can detect these impurities. **Methods:** We established and evaluated an analytical method using size-exclusion chromatography–multi-angle light scattering (SEC-MALS) for particle and protein impurity analyses of EV samples. **Results:** In the particle size distribution analysis of EV samples, SEC-MALS showed higher resolution compared with nanoparticle tracking analysis (NTA) and dynamic light scattering (DLS). MALS showed comparable accuracy and precision to that of other methods for particle size evaluation using polystyrene standard beads with 60, 100, or 200 nm diameter. Coupling SEC-MALS with UV detection quantitatively evaluated soluble protein impurities. Proteomic analysis on the SEC-MALS-fractionated samples identified different EV and lipoprotein marker proteins in different fractions. **Conclusions:** SEC-MALS can characterize EV preparations obtained from human adipose-derived mesenchymal stem cells, suggesting that it can evaluate the particle component composition in various EV samples and therapeutic exosome preparations.

## 1. Introduction

Extracellular vesicles (EVs) are submicrometer-sized biological particles secreted by nearly all cell types. Among these kinds of particles, exosomes generated from late endosomes have diameters ranging from 50 to 150 nm [1], transport mRNAs, miRNAs, and proteins from host cells to other cells and mediate various biological processes, including proliferation, immune responses, and cancer metastasis [2,3,4,5,6,7]. The cargo molecules and physiological functions of exosomes vary depending on the producing cells [8], and some types of exosomes have preferable effects on the human body. In particular, mesenchymal stem cell (MSC)-derived exosomes exert therapeutic effects in wound healing [9,10], stroke [11,12], and other diseases [13]. Therefore, research on their pharmaceutical applications is actively ongoing. In addition to native exosomes, engineered exosomes containing therapeutic oligonucleotides or other active substances have emerged in recent years [14,15,16]. Therefore, the number of clinical trials involving exosomes and other EV preparations has increased [17,18]. However, research on EVs and the development of EV-based pharmaceuticals face challenges in the characterization and quality control of therapeutic EV preparations containing exosomes and the establishment of suitable analytical methodologies.

Various isolation techniques (including ultracentrifugation, ultrafiltration, size exclusion chromatography, ion exchange chromatography, affinity chromatography, and others tailored to specific EV types) are utilized to purify EVs from cell culture supernatants and biological samples like serum and plasma [19,20]. However, these methods often encounter difficulties in effectively removing other EVs, lipoproteins, and soluble protein impurities that share similar attributes with the target EVs, such as particle size and density [21]. Although the potential impact of these impurities on the efficacy and safety of therapeutic EV preparations remains under investigation, it is necessary to rigorously assess their composition and content as potential critical quality attributes (CQAs) to ensure the quality of drug products.

Particle size is an important characteristic of exosomes that can be used as active pharmaceutical ingredients in EV preparations. As different types of EVs and lipoproteins often have slightly different sizes, analyzing the particle size distribution in an EV preparation is important to monitor particulate contaminants [22]. The techniques currently used to evaluate the size of biological nanoparticles include nanoparticle tracking analysis (NTA), dynamic light scattering (DLS), tunable resistance pulse sensing (TRPS), multi-angle light scattering (MALS), nano-flow cytometry, atomic force microscopy (AFM), electron microscopy (EM), and so on [23,24,25]. Among them, cryo-electron microscopy (cryo-EM) is the most precise sizing method, as described in Minimal Information for Studies of Extracellular Vesicles 2023 (MISEV2023) [20]; however, its limited accessibility and throughput are unsuitable for routine multi-sample measurements. Therefore, there is a demand for analytical methods that facilitate routine measurements on a larger number of samples [26], yet no consensus has been reached.

Among these nanoparticle measurement techniques, we anticipate that MALS combined with size-exclusion chromatography (SEC) could be a useful tool for the characterization and quality control of therapeutic EV preparations. MALS measures the scattered light generated by the laser irradiation of particles in solution from multiple angles, thereby enabling the determination of the particle radius of rotation based on the scattered light intensity patterns. Although MALS only provides comprehensive measurements of bulk particles, its integration with size exclusion high-performance chromatography (SEC-MALS) or field-flow fractionation (FFF-MALS) systems facilitates the calculation of average particle sizes within specific size ranges [27,28,29,30]. Importantly, these methodologies offer the unique advantage of fractionating and recovering individual particle populations based on their size, rendering them invaluable tools for quality evaluation. However, these methods are not as common as other size-determining methods; in particular, there are few reports on SEC-MALS performance and utility in EV measurement, despite the high prevalence of high-performance liquid chromatography (HPLC).

In addition to particle impurities, evaluating soluble protein impurities is necessary for EV formulations. The ratio of particle number to total protein (or its inverse) is often used as an indicator of the amount of protein impurities in EV formulations [21]. However, direct evaluation of the amount of soluble proteins separated from EV-derived proteins is not possible using conventional protein quantification methods. Hence, there is a need for a simple high-throughput analytical method to evaluate soluble protein impurities in EV samples.

Since the particle size distribution is crucial to understand the physicochemical properties and purity of therapeutic EV preparations, we aimed to establish the SEC-MALS method and evaluate its performance for the characterization of EV preparations (particularly for samples containing heterogeneous particles and other impurities), considering the various situations expected in the development of therapeutic EV preparations. We also investigated the potential of SEC-MALS to assess the amount of protein impurities and the composition of particulate impurities in the EV samples.

## 2. Materials and Methods

### 2.1. Extracellular Vesicle Preparation

Extracellular vesicle samples were prepared from the A549 cell supernatant. A549 cells were seeded into 150 cm^2^ tissue culture flasks (Iwaki, Shizuoka, Japan) and cultured in Dulbecco’s modified Eagle’s medium (DMEM) supplemented with 1× penicillin/streptomycin and 10% inactivated fetal bovine serum (FBS). At 50% confluency, the cells were washed with Dulbecco’s Phosphate-Buffered Saline (D-PBS) and incubated with 50 mL of OPTI-MEM (Thermo Fisher Scientific, Waltham, MA, USA) at 37 °C under 5% CO_2_ for 48 h. The conditioned OPTI-MEM was collected, centrifuged at 2000× *g* for 10 min, and the supernatant was filtered with a 0.22 μm filter unit. The filtered medium was concentrated with 100 K MWCO ultrafiltration units (Amicon Ultra, Merck Millipore, Burlington, MA, USA) to approximately 1/100 volume (crude sample) and filtered through a 0.22 μm syringe filter (Milex GV, Merck Millipore, Darmstadt, Germany). The crude sample buffer was replaced with D-PBS (UF sample) and separated into three aliquots. One of the aliquots was purified by a size-exclusion gravity column (Pure-EVs, Hansabio, Tallinn, Estonia) following the user’s guide and the 7th fraction (approximately 3.5–4.2 mL from the initial point) was used as the SG sample. The PS sample was prepared from an aliquot using phosphatidylserine affinity beads (MagCapture Exosome Isolation Kit PS Ver.2, FujifilmWako, Tokyo, Japan). All samples were stored at 4 °C until subsequent measurements. The EV samples derived from human adipose-derived mesenchymal stem cells (hAdMSCs) were purchased from CellSource Co., Ltd. (Tokyo, Japan), which were purified from cell-culture supernatant of hAdMSCs by centrifugation, filtration, and tangential flow filtration (TFF). During TFF, the buffer was exchanged with PBS and condensed to the following concentration factors: Batch A ×30; Batch B ×0 (no condensation) and ×300; and Batch C ×0, ×30, and ×150. Prior to conducting relevant experiments, the use of hAdMSC-derived EV samples was approved by the Institutional Research Ethics Committee (Application No. 359).

### 2.2. Nanoparticle Tracking Analysis (NTA)

Nanoparticle tracking analysis measurements were performed using a Nanosight NS300 (Malvern Panalytical, Malvern, UK) with a 532 nm laser and analyzed by NTA 3.4—Sample Assistant Build 3.4.4 software (Malvern Panalytical). Before each measurement, the system performance was tested using 100 nm standard beads according to the manufacturer’s instructions. The NTA samples were prepared at the following concentrations by stepwise dilution: 1 × 10^7^–1 × 10^9^ particles/mL for standard-sized particles and 3.13 × 10^7^–5.00 × 10^9^ particles/mL for EV samples. The camera level was adjusted for each sample during each measurement, and video data were acquired 3 times (for size standard particles) or 5 times (for EV samples) for 60 s in continuous flow using a syringe pump set at a speed of 40. For polystyrene-sized standard particles (Nanosphere Size Standards: 60, 100, and 200 nm; Thermo Fisher Scientific), the camera levels were automatically optimized depending on their size and concentration (60 nm: 16, 100 nm: 11–16, and 200 nm: 6–8). Meanwhile, the camera level for all EV samples was uniformly set to 16. All measurements were repeated three times and the average diameter was evaluated.

### 2.3. Dynamic Light Scattering (DLS)

Dynamic light scattering size measurements were performed using a Zetasizer Nano ZS instrument (Malvern Panalytical). The system was calibrated using 60 nm and 200 nm polystyrene size standard particles at a concentration directed by the manufacturer’s manual. The DLS samples were adjusted to the following concentrations: 1 × 10^7^–1 × 10^9^ particles/mL for standard-sized particles and 1.25 × 10^8^–2.00 × 10^9^ particles/mL for EV samples. The number of runs in each measurement was automatically optimized based on the scattering intensity of the sample. All measurements were performed three times and the average diameter and polydispersity (PDI) were calculated.

### 2.4. Size Exclusion HPLC with Multi-Angle Light Scattering (SEC-MALS)

Each sample was separated using HPLC (ACQUITY Premier, Waters, Milford, MA, USA) equipped with a size-exclusion column (Shodex OHpak SB-806 HQ, Rezonac, Tokyo, Japan) and a target molecular weight ranging from 100,000 to 20,000,000. The eluted samples were analyzed using an in-line multi-angle light-scattering detector (DAWN NEON, Wyatt Technologies, Goleta, CA, USA) with a laser at 658 nm. Further, 10 μL standard particles or 100 μL EV samples were injected into the SEC-MALS system and eluted at a flow rate of 1 mL/min. The mobile phase was 10 mM NaCl for polystyrene size standards and D-PBS for EV samples. Data acquisition and analysis were performed using Wyatt’s ASTRA software ver.8.1.0.18 and the mean radius was calculated by fitting the data collected from 18 different angles to the “sphere” model algorithm in the software. A photodiode array (PDA) detector was connected to the SEC-MALS system, and absorbance data were collected at 280 nm.

### 2.5. Characterizing SEC-MALS Fractions

The crude sample (100 μL) was analyzed using SEC-MALS with tandemly connected double SP-806 HQ columns and the elution of each peak was collected in a 15 mL centrifugal tube. The collected fractions were analyzed using NTA and CD9/CD63 sandwich enzyme-linked immunosorbent assay (ELISA) kits (CD9/CD63 Exosome ELISA Kit; Cosmo Bio, Tokyo, Japan). For proteomic analysis, the fractions were concentrated using a 100 K molecular weight cut-off (MWCO) ultrafiltration unit and the protein concentration of each fraction was measured using a Qubit protein assay (Thermo Fisher Scientific). Proteomic analysis was performed using condensed sample solutions corresponding to 2 μg of protein, according to a previously reported method [31]. Briefly, each sample solution was treated with the MPEX PTS reagent (GL Sciences, Tokyo, Japan) for reduction and carboxymethylation. The treated samples were digested using 1 μg of trypsin and 1 μg of trypsin/Lys-C mixtures at 37 °C for 16 h. The digested peptides were desalted using an Oasis HLB μElution plate (Waters), dried, and dissolved in 15 μL of 0.1% formic acid solution. The prepared samples were analyzed by liquid chromatography–tandem mass spectrometry (LC-MS/MS) using an UltiMate 3000 RSLCnano LC system with an Easy-Spray LC column and Orbitrap Fusion Lumos Tribrid mass spectrometer (Thermo Fisher Scientific). The mobile phase was 0.1% formic acid and 0.1% formic acid in acetonitrile (buffer A). The peptides were eluted at a flow rate of 300 nL/min with a gradient of 2–40% buffer A over 45 min. The MS/MS conditions were as follows: an electrospray voltage of 2.0 kV in positive ion mode, a capillary temperature of 250 °C, and a collision energy of 28%. The spectral data obtained by LC-MS/MS were subjected to database search analysis using the SEQUEST algorithm (Proteome Discoverer 2.4.1.15, Thermo Fisher Scientific) using the UniProt database. Static carboxymethylation (58.0 u) at Cys was used as a modified parameter for database search analysis.

## 3. Results

### 3.1. Evaluating the Usefulness of SEC-MALS in Measuring Particle Size Distribution of EV Samples

**Figure 1:**
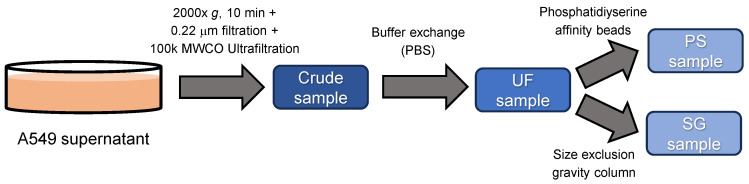
A549-devived EV preparation for DLS, NTA, and SEC-MALS. The A549 culture supernatant was condensed by ultrafiltration (crude sample), followed by buffer exchange with PBS (UF sample). The UF samples were purified using phosphatidylserine affinity beads (PS sample) and a size-exclusion gravity column (SG sample).

**Figure 2:**
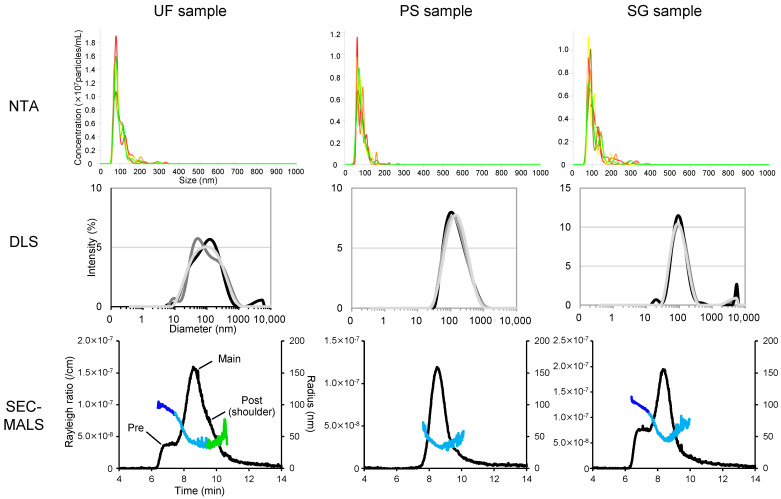
Evaluating EV preparations by NTA, DLS, and SEC-MALS. Size distribution analysis of the three EV samples using NTA, DLS, and SEC-MALS. Five runs in each NTA measurement and three runs in each DLS measurement were depicted in different colors (for NTA measurement, 1st: red, 2nd: orange, 3rd: yellow, 4th: dark green, and 5th: light green; for DLS measurement, 1st: black, 2nd: dark gray, and 3rd: light gray). The colored plots on the MALS chromatograms indicate the calculated radii of gyration (blue: pre-peak, light blue: main peak, green: post-peak).

We first assessed the usefulness of SEC-MALS to evaluate particulate composition in EV samples, which were assumed to be therapeutic EV preparations, by comparing it with NTA and DLS, which are commonly used for EV measurement. EV samples used in this evaluation were prepared with different purities. UF samples were prepared by ultrafiltration and buffer exchange from the A549 cell culture supernatant, and PS and SG samples were purified from UF samples using phosphatidylserine affinity beads or a size exclusion gravity column (Figure 1). First, each sample was analyzed using NTA, DLS, and SEC-MALS to examine whether differences in mean particle size, particle size distribution, and EV composition owing to differences in purification methods could be detected (Table 1 and Figure 2).

In the NTA analysis, shoulder peaks on the main peak and some small peaks of 200 nm or larger were observed in the UF and SG samples that were not present in the PS sample. However, there was only a slight difference in the overall shape of the particle size distribution charts among the three samples. The relative standard deviation (RSD) of diameter was ≤7.7% for all samples, and the mean particle size varied between 101.3 nm for the UF sample, 90.7 nm for the PS sample, and 110.1 nm for the SG sample. Given these results, the mean particle size can be measured with high precision using NTA, and the differences between samples can also be assessed.

In the DLS analysis, the peak of the size distribution chart was broader than that of the NTA, and the reproducibility of the particle size distribution chart for the UF sample was poor, which cast doubt on the accuracy of the measured particle size. The accuracy of the DLS measurement was worse than that of NTA, with an RSD of 14.9% for the PS sample. The UF sample had a low RSD and there was a difference of over 30 nm in the mean particle size compared to the other methods. In contrast, the PDI was smaller for the PS and SG samples than for the UF sample based on the DLS measurements with a lower degree of purification (Supplementary Table S1), suggesting that the relative degree of purification using DLS can be approximated.

The SEC-MALS analysis yielded chromatograms with distinct shapes for each sample. Three peaks were observed for the UF sample, including a shoulder peak, two peaks were observed for the SG sample, and one peak was observed for the PS sample (Figure 2). This showed that SEC-MALS most clearly visualized the different compositions of EV samples with different purification methods compared to the other two analytical methods. The calculated particle sizes for each peak revealed different average particle sizes in the main, pre-peak, and post-peak phases (Supplementary Table S1). The maximum RSD of the calculated mean diameter of the main peak was 4.6% (Table 1), demonstrating a precision comparable to that of NTA and highlighting the accurate analytical capability of MALS (Table 1). Notably, the SEC-MALS chromatograms also exhibited a distinct pre-peak containing particles approximately 200 nm in the UF and SG samples, where peaks were observed at sizes above 200 nm in NTA, suggesting that SEC-MALS can reflect the difference in large particulate content among samples more clearly than NTA.

Subsequently, we conducted a dilution series analysis for each sample using each method to ascertain the impact of sample concentration on the measured particle size and accuracy (Supplementary Figure S1 and Table S1). The dilution of samples for NTA resulted in a minor reduction in the analysis accuracy, whereas the particle size measurement remained relatively unaffected, and consistent values were calculated. DLS measurements showed that the effect of dilution was greater than that of NTA, with a maximum change in particle size of >20 nm and an increase in RSD and PDI. A concentration of at least 10^9^ particles/mL was considered necessary for the DLS measurement because the measurement of the lowest concentration of PS samples sometimes interrupted the analysis owing to insufficient intensity of the scattered light. In SEC-MALS, there was a significant increase in the particle size of the main peak and sub-peak, as well as a decrease in accuracy in measurements with a minimum concentration of 2.5 or 5 × 10^8^ particles/mL. A sample with a concentration of 1 × 10^9^ particles/mL or higher was considered preferable for proper particle size evaluation using SEC-MALS because these results were considered to be caused by a decrease in the intensity of the scattered light, as in DLS.

### 3.2. Evaluating Analytical Performance Using Standard Particles

We attempted to compare the particle size analysis capabilities and characteristics of SEC-MALS with those of NTA and DLS to evaluate the performance of SEC-MALS in measuring particle size distribution. Standard polystyrene particles (60, 100, and 200 nm) were used as analytes and the accuracy and precision of the particle diameters were evaluated from the measurement results of the dilution series for each particle (Table 2). However, the column was removed from the HPLC system and the performance of MALS alone was evaluated because these standard particles could not be analyzed by SEC-MALS owing to adsorption onto the SEC column. The appropriate sample concentration differs depending on the measurement method used. At high concentrations, the overlapping of particles was observed in the captured video on NTA and saturation of the scattered light intensity was observed in MALS (Supplementary Figure S4). On the other hand, the intensity of scattered light was reduced in DLS and MALS at low concentrations, making them insufficient for measurement, and the influence of noise was significant, resulting in a deterioration of accuracy and a decrease in reproducibility (Supplementary Figures S3 and S4, Table 2).

Among the three methods, NTA showed good analytical performance regardless of the particle size at concentrations below 1 × 10^9^ particles/mL. Although some sub-peaks appeared in the particle size distribution at low concentrations, the measured mean diameters had an accuracy of 94.1% and a maximum RSD of 6.5% (Supplementary Figure S2).

In the DLS analysis, the particle size affected the analytical performance. For the 60 nm standard particles, accuracy and precision began to deteriorate at 1 × 10^9^ particles/mL and the accuracy increased to 154.4% at 1 × 10^8^ particles/mL, whereas the precision deteriorated to 15%. The PDI was 0.316, indicating that the sample was polydispersed at this concentration. However, the accuracy and precision were equivalent to those of NTA for samples with concentrations of 1 × 10^8^ particles/mL or higher for the 100 nm and 200 nm standard particles, and the PDIs were <0.05, correctly reflecting the fact that the samples were monodisperse.

In MALS measurements, differences in the analytical performance based on particle size were similar to that for DLS. For 60 nm particles, MALS tended to overestimate the particle size with an accuracy of 133% compared to the actual size, even in the sample with the highest concentration of 1 × 10^11^ particles/mL. Furthermore, the accuracy and precision deteriorated at lower concentrations. Meanwhile, the accuracy for 100 nm particles was within 100 ± 10% and the RSD was lower than 3% for the samples with 1 × 10^9^ particles/mL or higher. The accuracy for 200 nm particles was within 100 ± 10% and the RSD was lower than 1.3% for samples with 1 × 10^7^ particles/mL or higher.

These results showed that MALS was possible to measure the diameter of particles larger than approximately 100 nm, with performance comparable to that of NTA and DLS by setting an appropriate concentration range; the particle size tended to be overestimated for smaller particles of approximately 60 nm. However, it is possible that slight changes in the solvent composition during injection may have a greater effect on the analysis of smaller particles with lower scattered light intensity because no fractionation by SEC was performed in this evaluation.

### 3.3. Evaluating Protein Impurities in EV Samples by SEC-UV-MALS

**Figure 3:**
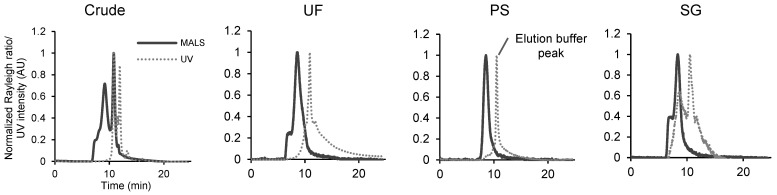
Size exclusion chromatography–ultraviolet MALS (SEC-UV-MALS) evaluation of protein impurities in EV samples. SEC-UV-MALS chromatograms of A549-derived crude, UF, PS, and SG samples. The MALS chromatograms are depicted by solid lines and the UV chromatograms are depicted by dotted lines.

We next examined the feasibility of purity evaluation using SEC-UV-MALS, which combines SEC-MALS with UV detection (Figure 3). For the crude, UF, and SG samples, UV absorption was confirmed at a later retention time than the MALS peak, demonstrating the successful separation of particle components and soluble protein impurities via SEC. For the PS sample, a prominent peak originating from the elution buffer of affinity beads appeared in the UV chromatogram, hindering proper evaluation. In crude and UF samples, the UV peak at the same retention time as particles was scarcely observed, and the majority of UV absorption was attributed to protein impurities where the particle numbers per protein (µg) were low (9.60 × 10^7^ and 9.36 × 10^7^ particles/μg protein, respectively) owing to limited purification. Conversely, a large UV peak, with its magnitude comparable to that of the protein impurity-derived peak was accompanied by the main peak of the MALS chromatogram in the well-purified SG sample with a higher particle number/protein ratio (2.70 × 10^8^ particles/µg protein). Although quantitative evaluation of the actual protein content percentage is difficult owing to the incomplete separation of the particle component from the protein impurity and the unknown response factor of UV absorption by the EV-derived protein, SEC-UV-MALS was considered useful to qualitatively evaluate the protein impurity content and monitor the degree of purification from proteins.

### 3.4. Detailed Characterization of Size-Dependently Isolated Fractions by SEC-MALS

**Figure 4:**
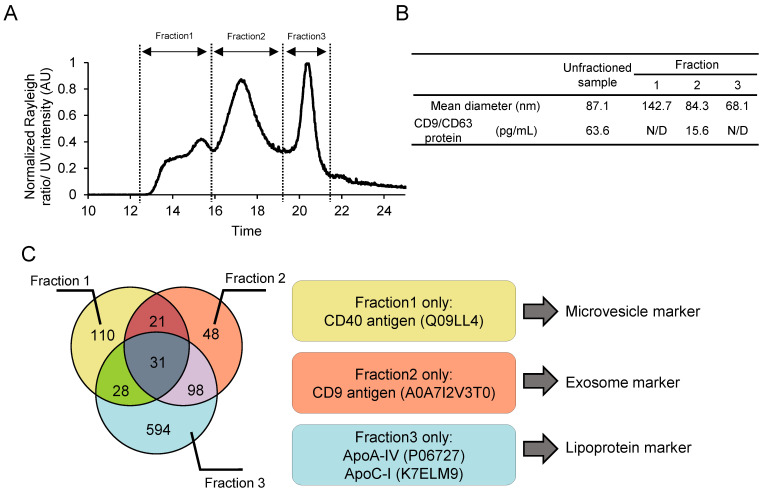
Size-dependent fractionation by SEC-MALS and precise characterization of particulate impurities. (**A**) Size-dependent fractionation of the A549 supernatant (crude sample). (**B**) A CD9/CD63 ELISA was performed to confirm the presence of exosomes in each fraction. (**C**) Proteomic analysis of the size-dependent isolated SEC-MALS fractions. The marker proteins of EVs and lipoproteins listed in ExoCarta or previous reports [32,33] are indicated from the detected peptides only contained in each fraction.

An advantage of SEC-MALS over other particle size analysis methods is that the particle population can be fractionated according to size and separately recovered. Using this advantage, we investigated whether SEC-MALS could identify the main constituent particle types of each fraction of an EV sample by fractionating and recovering the particulate components of a coarse EV sample by size and characterizing them in detail. Tandemly connected size exclusion columns were used for fractionation by SEC-MALS (Figure 4A), and each peak in the chromatogram was collected as fractions 1–3. The average particle size of each fraction was analyzed using NTA as follows: fraction 1: 142.7 nm, fraction 2: 84.3 nm, and fraction 3: 68.1 nm (Figure 4B), indicating that the particles in the sample were successfully fractionated according to their size and collected. The first step of characterization utilizing sandwich ELISA with CD9/CD63 for these fractions showed a positive signal in fraction 2, strongly suggesting that this fraction contained exosomes and the other fractions were mainly composed of other particles. To identify the major components of fractions 1–3, each fraction was subjected to proteomic analysis. The number of unique proteins identified in each fraction was as follows: fraction 1: 110; fraction 2: 48; and fraction 3: 594. We searched for various EV markers among the proteins contained in each fraction from ExoCarta (the exosome database) and previous reports [32,33] and found that fraction 1 contained CD40, which is considered as a microvesicle marker; fraction 2 contained CD9, a well-known exosome marker; and fraction 3 contained ApoA-IV and ApoC-1, which are components of lipoproteins (Figure 4C), implying that each fraction is composed of different types of particles. Each fraction also contains several types of apolipoproteins; therefore, lipoproteins may be present in all fractions. This does not contradict the fact that lipoproteins have a wide range of size distributions.

These results indicate that SEC-MALS can be used to evaluate the particulate components in EV samples by combining fractionation using SEC-MALS with characterization techniques, such as ELISA and proteomics, and that SEC-MALS is useful to characterize the particulate and other protein components in EV preparations.

### 3.5. SEC-MALS Application for the Quality Evaluation of hAdMSC-Derived EVs

**Figure 5:**
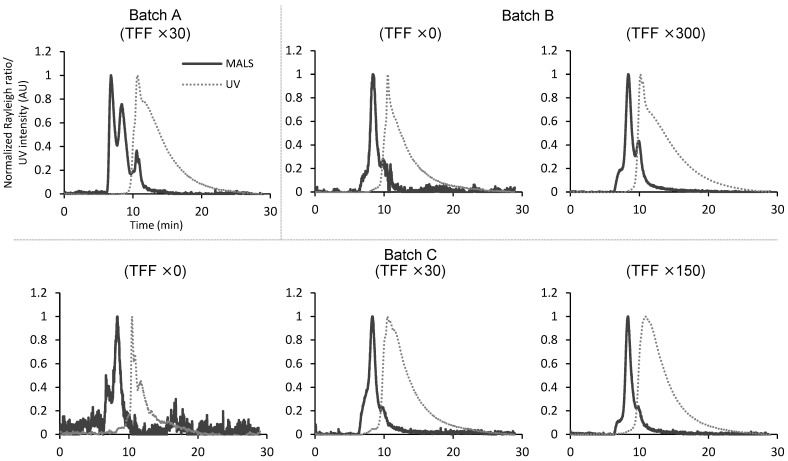
Size exclusion chromatography–UV-MALS evaluation of hAdMSC-derived EVs. Chromatograms of three batches of hAdMSC-derived EV (batches A, B, and C). The MALS chromatograms are depicted by solid lines and the UV chromatograms are depicted by dotted lines. The tangential flow filtration (TFF) concentration rate of each sample is shown in the chromatograms.

Finally, we investigated whether the changes in particle composition and protein impurity content between different batches of hAdMSC-derived EV preparations, which have been intensively studied for pharmaceutical applications in recent years, could be assessed using SEC-MALS (Figure 5). For this purpose, three batches of EV samples (batches A, B, and C) produced from different host cells using different purification processes were used.

Among the three batches, batch A showed a distinctly different MALS chromatogram from batch C and contained more larger-sized particles than the other samples. In batch A, unsorted hAdMSCs were used for EV preparation, whereas sorted cells were used for batch C preparation. Therefore, this variation in particle composition was likely owing to differences in the producing cells, suggesting that SEC-MALS can detect differences in particle composition in EV samples produced from different host cells. The MALS chromatograms showed that the content of smaller-sized particles eluted later than the main peak increased in batches B and C as enrichment progressed. This result contradicts the TFF principle of filtering soluble components and small particles into the pores and suggests that particles in the main peak are more likely to adsorb to the container and collapse during TFF than small particles, resulting in a change in particle composition. However, it was difficult to identify peaks at the same elution time as the main peak in the MALS chromatograms when the content of protein impurities in each sample was assessed from the UV chromatograms, even in the highly concentrated 150× and 300× samples, which can be observed in the well-purified SG samples, and most proteins were present as soluble components. This result indicated that a relatively high amount of protein impurities remained after TFF purification and SEC-MALS was useful to characterize these EV samples.

## 4. Discussion

In this study, we established the SEC-MALS analysis method and evaluated its performance in measuring particle size distribution in comparison with NTA and DLS by analyzing standard particles and EV preparations. For the particle size measurement of monodisperse standard particles, NTA showed better accuracy and precision because it can measure individual scattered light to trace the Brownian motion of individual particles, whereas MALS and DLS measure the pattern or intensity of scattered light from bulk solutions. However, SEC-MALS showed the most obvious difference in the size distribution of particulate components in the measurement of polydisperse EV samples among the EV samples, owing to the size-dependent separation by SEC, whereas NTA displayed only a slight difference among all samples. Fractionation by SEC is useful for particle-to-particle separation and for particle-to-protein component separation and enables the direct assessment of soluble proteins in EV samples prepared using different manufacturing profiles.

Taking advantage of the recoverability of the fractionated samples, we demonstrated that the fractionation using SEC-MALS is useful to identify the types of particulate components in EV samples. Although this study does not clarify whether each A549 cell releases these particles simultaneously or there are several subpopulations of A549 cells in our culture system which release different types and ratios of EVs, the SEC-MALS method may also enable efficient screening of the production cell clones that release the target type of EVs for therapeutic purposes. We focused on the marker proteins of each fraction in our proteomic analysis, but it should also be possible to perform more detailed particle profiling using glycomics and lipidomic analyses, which could be useful to identify CQAs for pharmaceutical activity.

Furthermore, SEC-UV-MALS demonstrated separation capabilities of hAdMSC-derived EV samples in terms of particle composition and protein impurities between different batches and concentration levels, where distinctions may not be clearly evident based solely on NTA analysis or protein quantification. Currently, well-developed methods for assessing soluble protein content in EV samples are lacking. In this context, SEC-UV-MALS analysis can be considered a valuable approach that provides convenient information on protein impurities. Based on these SEC-UV-MALS results, we propose a quantification method for EVs that can be employed in experiments and clinical administration. Traditionally, EV quantities are assessed based on either particle number or protein mass. However, our SEC-UV-MALS measurements revealed significant variations in the protein content depending on the manufacturing method used. Consequently, defining dosages based on particle number is deemed more desirable when considering the clinical application of EV formulations. Meanwhile, the extent to which proteins should be removed from EV formulations should continue to be examined, since some reports suggest that EVs and available proteins exert their effects in a co-operative manner [34], with a focus on efficacy and safety.

As described thus far, SEC-MALS is a prominent approach to evaluate EV samples. However, the performance evaluation using standard particles in this study could only be performed for MALS owing to particle adsorption on the SEC column and could not be performed under the same conditions as the SEC-MALS analysis of the EV samples. Therefore, it is necessary to develop standard particles composed of components similar to EVs with appropriately assigned diameters to evaluate the performance of SEC-MALS more appropriately in the future.

Several novel techniques such as nano-flow cytometry and super-resolution microscopy have been in the spotlight in addition to MALS, NTA, and DLS. These techniques have different measurement principles and characteristics. Therefore, it is important to use multiple techniques that are suitable for the intended purpose of appropriate evaluation of nanoparticles with an understanding of the characteristics of each analytical method. In this regard, SEC-MALS is expected to be a promising tool as one of these techniques.

## Figures and Tables

**Table 1:** Summary of particle size measurements of EV samples.

Sample ^a^	UF		PS		SG	
Method	Diameter ^b^ (nm)	RSD (%)	Diameter ^b^ (nm)	RSD (%)	Diameter ^b^ (nm)	RSD (%)
NTA	101.3	2.8	90.7	7.7	110.1	0.9
DLS	67.0	3.2	96.4	14.9	101.7	2.4
SEC-MALS	97.4 ^c^	3.9	76.2 ^c^	4.6	112.7 ^c^	0.7

^a^ The sample concentration for each measurement method was as follows: 5.00 × 10^8^ particles/mL for NTA, 2.00 × 10^9^ particles/mL for DLS, 4.00 × 10^9^ particles/mL for SEC-MALS. ^b^ Each value indicates an average of three measurements. ^c^ Mean diameter of main peak. Abbreviations: DLS, dynamic light scattering; NTA, nanoparticle tracing analysis; SEC-MALS, size exclusion high-performance chromatography with multi-angle light scattering; PS, phosphatidylserine sample; SG, size-exclusion gravity column sample; UF, ultrafiltration sample; RSD, relative standard deviation.

**Table 2:** Summary of particle size measurements of standard particles using NTA, DLS, and MALS.

**NTA**	**Std. Particles**	**60 nm ^a^**				**100 nm ^a^**				**200 nm ^a^**			
	**Concentration ^c^** **(Particles/mL)**	**Diameter (nm) ^b^**	**Accuracy (%)**	**RSD (%)**		**Diameter (nm) ^b^**	**Accuracy (%)**	**RSD (%)**		**Diameter (nm) ^b^**	**Accuracy (%)**	**RSD (%)**	
	1 × 10^9^	61.3	98.9	0.2		95.0	94.1	0.1		193.0	95.1	0.3	
	1 × 10^8^	62.6	101.0	4.0		95.6	94.7	1.5		195.5	96.3	0.6	
	1 × 10^7^	65.3	105.3	0.8		99.4	98.4	2.9		197.4	97.2	6.5	
**DLS**	**Std. Particles**	**60 nm ^a^**				**100 nm ^a^**				**200 nm ^a^**			
	**Concentration** **(Particles/mL)**	**Diameter (nm) ^b^**	**Accuracy** **(%)**	**RSD** **(%)**	**PDI**	**Diameter** **(nm) ^b^**	**Accuracy** **(%)**	**RSD** **(%)**	**PDI**	**Diameter** **(nm) ^b^**	**Accuracy** **(%)**	**RSD** **(%)**	**PDI**
	1 × 10^11^	65.0	104.8	0.5	0.011	102.6	101.6	0.6	0.014	205.9	101.4	0.3	0.014
	1 × 10^10^	66.5	107.3	0.2	0.020	103.4	102.4	1.6	0.011	206.2	101.6	0.9	0.015
	1 × 10^9^	69.5	112.1	4.0	0.088	104.5	103.5	3.6	0.010	205.7	101.3	0.0	0.010
	1 × 10^8^	95.7	154.4	15.0	0.316	106.9	105.8	4.5	0.043	209.3	103.1	1.0	0.029
	1 × 10^7^	- ^d^				115.6	114.5	6.3	0.275	237.7	117.1	10.3	0.153
**MALS**	**Std. Particles**	**60 nm ^a^**				**100 nm ^a^**				**200 nm ^a^**			
	**Concentration** **(Particles/mL)**	**Diameter (nm) ^b^**	**Accuracy (%)**	**RSD (%)**		**Diameter (nm) ^b^**	**Accuracy (%)**	**RSD (%)**		**Diameter (nm) ^b^**	**Accuracy (%)**	**RSD (%)**	
	1 × 10^11^	82.5	133.1	8.8		94.5	93.6	2.3		- ^e^			
	1 × 10^10^	91.3	147.3	8.2		103.7	102.7	2.0		208.9	102.9	0.6	
	1 × 10^9^	117.0	188.7	7.2		110.7	109.6	2.9		208.6	102.8	0.6	
	1 × 10^8^	164.8	265.8	21.5		116.2	115.0	9.3		209.3	103.1	1.3	
	1 × 10^7^	227.1	366.3	21.0		269.5	266.8	12.8		214.7	105.8	0.5	

^a^ The certified diameters of the standard particles were 62, 101, and 203 nm. ^b^ The values indicate the average of three measurements. ^c^ The particle density was too high for the observation of individual particles in the NTA analysis. ^d^ The intensity of the scattered light was inadequate for the DLS measurements. ^e^ The intensity of the scattered light was saturated on the MALS chromatogram and the proper diameter could not be calculated. PDI, polydispersity index.

## Data Availability

All data acquired or analyzed in this study are included in the published article and its Supplementary Materials. Data and experimental materials are available from the corresponding author upon reasonable request.

## Supplementary Materials

The following supporting information can be downloaded at: https://www.mdpi.com/article/10.3390/pharmaceutics16121526/s1, Figure S1: Impact of dilution on nanoparticle tracking analysis (NTA), dynamic light scattering (DLS), and size-exclusion chromatography–multi-angle light scattering (SEC-MALS); Figure S2: Nanoparticle tracking analysis measurements of size standard particles; Figure S3: Dynamic light scattering measurements of size standard particles; Figure S4: Multi-angle light scattering (MALS) measurements of size standard particles; Table S1: Effect of dilution on particle size measurements of EV samples.
