# Supplementary material for: Usefulness of Size-Exclusion Chromatography–Multi-Angle Light Scattering to Assess Particle Composition and Protein Impurities for Quality Control of Therapeutic Exosome Preparations
Source: Pharmaceutics. 2024 Nov 27;16(12):1526. doi: 10.3390/pharmaceutics16121526 (PMC11728667; doi:10.3390/pharmaceutics16121526)
Supplement: Supplementary file 1 [file pharmaceutics-16-01526-s001.zip › pharmaceutics-3315158-supplementary.pdf]

Supplementary Material

Usefulness of Size-Exclusion Chromatography–Multi-Angle Light Scattering to Assess Particle Composition and Protein Impurities for Quality Control of Therapeutic Exosome Preparations

Hirotaka Nishimura [1], Noritaka Hashii [1], Tomofumi Yamamoto [1], Yuchen Sun [2], Takumi Miura [3], Yoji Sato [4], and Akiko Ishii-Watabe [1,*]

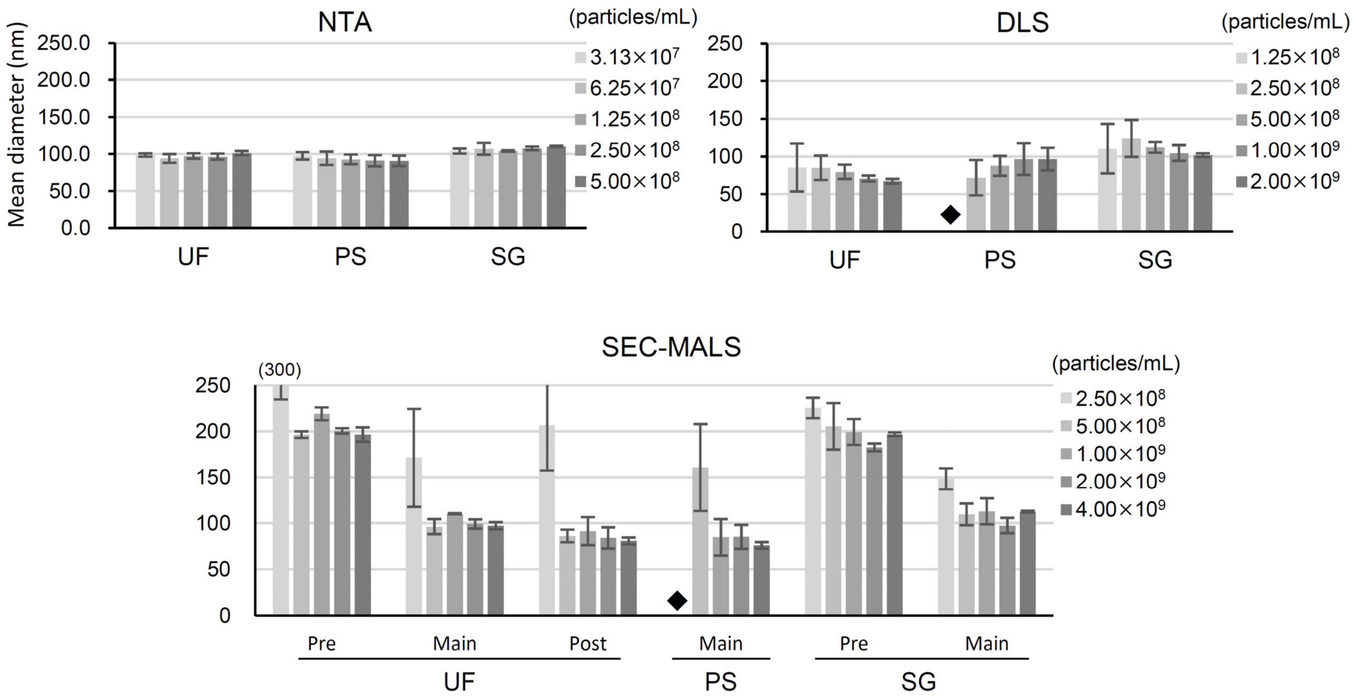

Supplementary Figure S1. Impact of dilution on nanoparticle tracking analysis (NTA), dynamic light scattering (DLS), and size-exclusion chromatography-multi-angle light scattering (SEC-MALS) for EV samples. Each measurement was repeated three times and the error bars represent the standard deviation. The PS could not be measured by NTA and SEC-MALS at the lowest concentration (◆). PS, phosphatidylserine affinity beads sample; SG, size exclusion gravity column sample; UF, ultrafiltration sample.

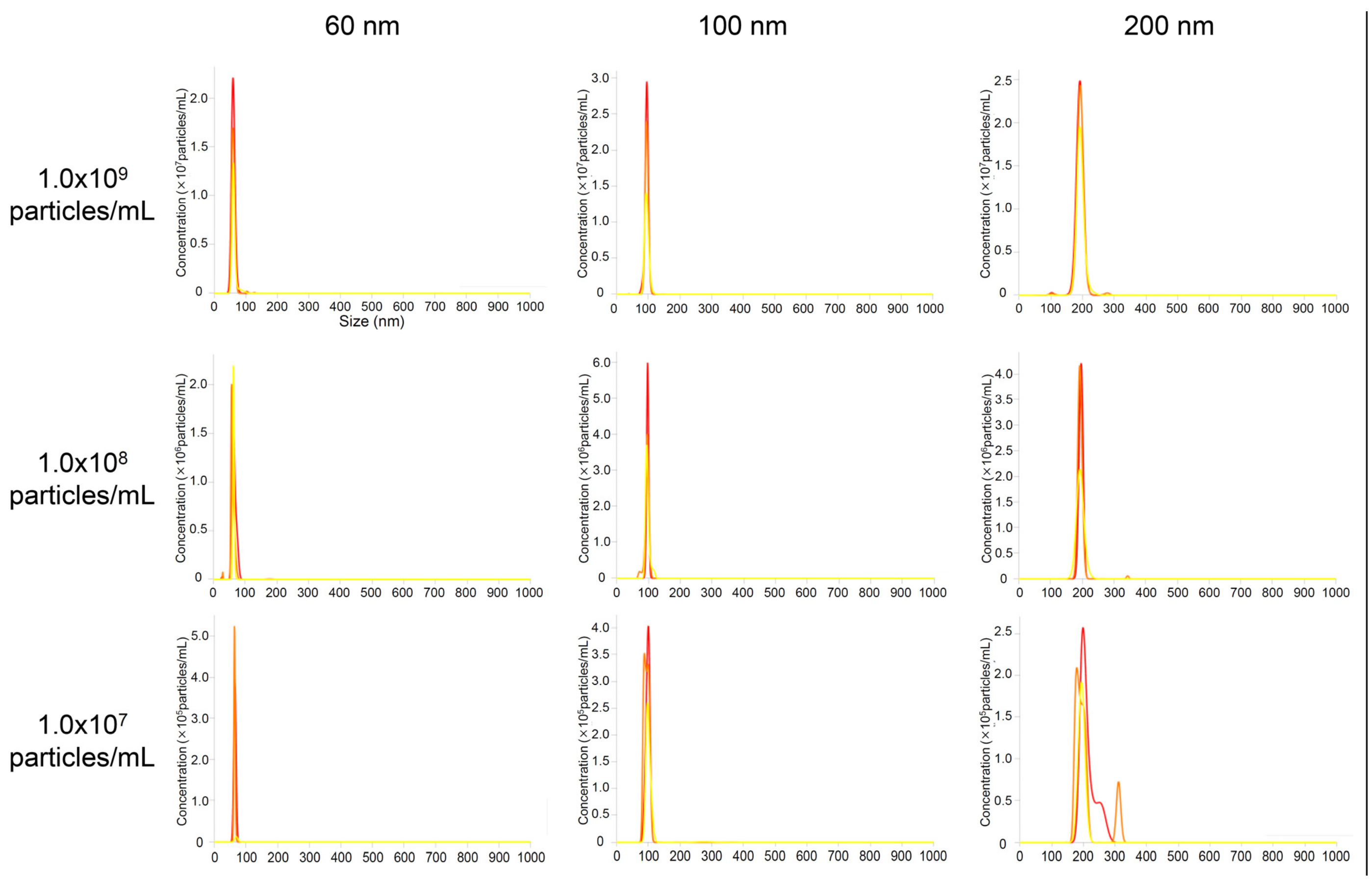

Supplementary Figure S2. Nanoparticle tracking analysis measurements of size standard particles. The particle size distributions of 60, 100, and 200 nm standard particles were measured at each particle concentration. Three runs in each measurement were depicted in different colors (1st: red, 2nd: orange, 3rd: yellow). The measurements were repeated three times and the mean diameter and RSD were used to evaluate the NTA performance on the particle size measurement.

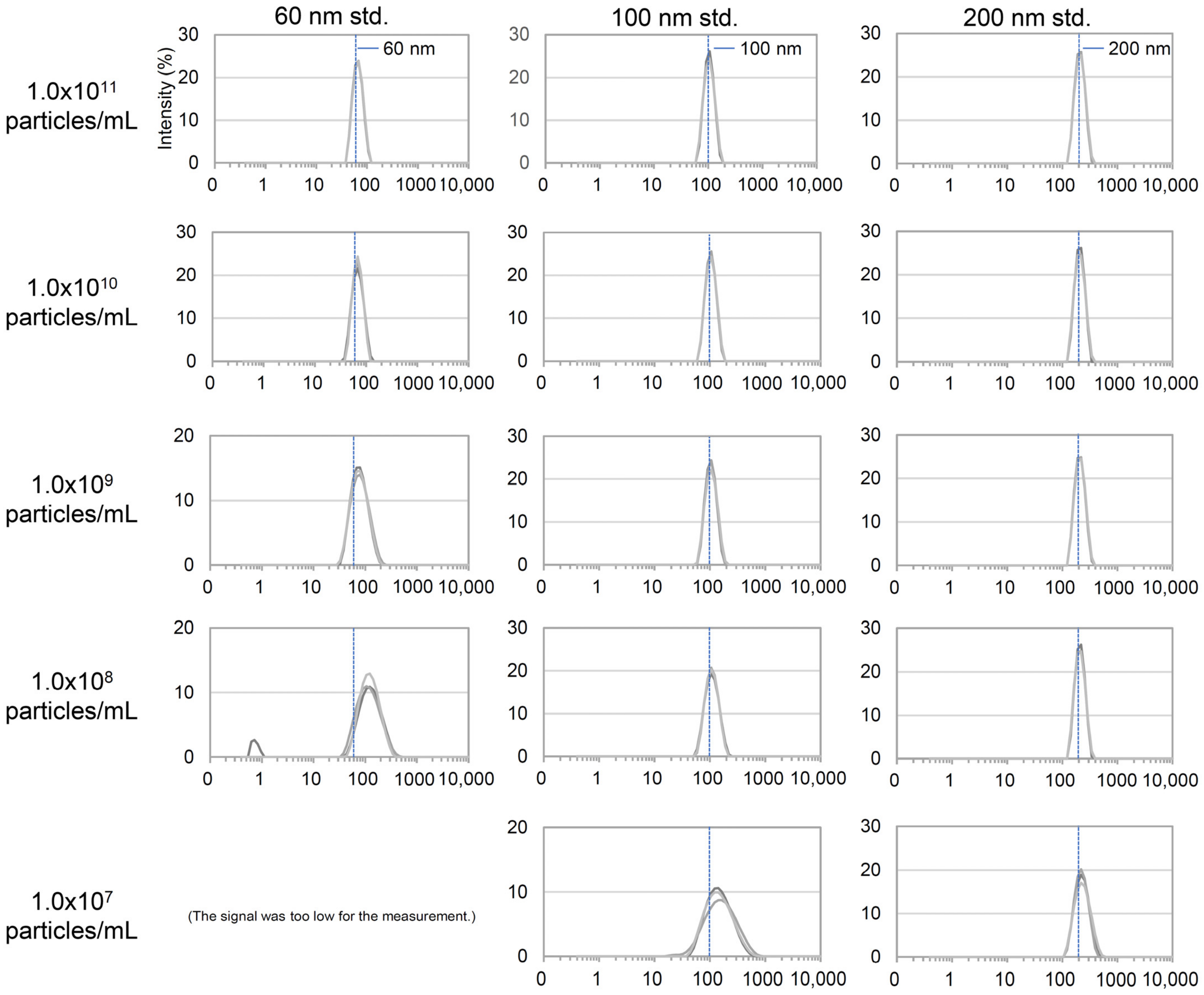

Supplementary Figure S3. Dynamic light scattering measurements of size standard particles. The particle size distributions of the 60, 100, and 200 nm size standard particles were measured by DLS at each concentration. Three runs in each measurement were depicted in different colors (1st: brack, 2nd: dark gray, 3rd: light gray). The measurements were repeated three times and the mean diameter and RSD were used to evaluate the DLS performance on the particle size measurement.

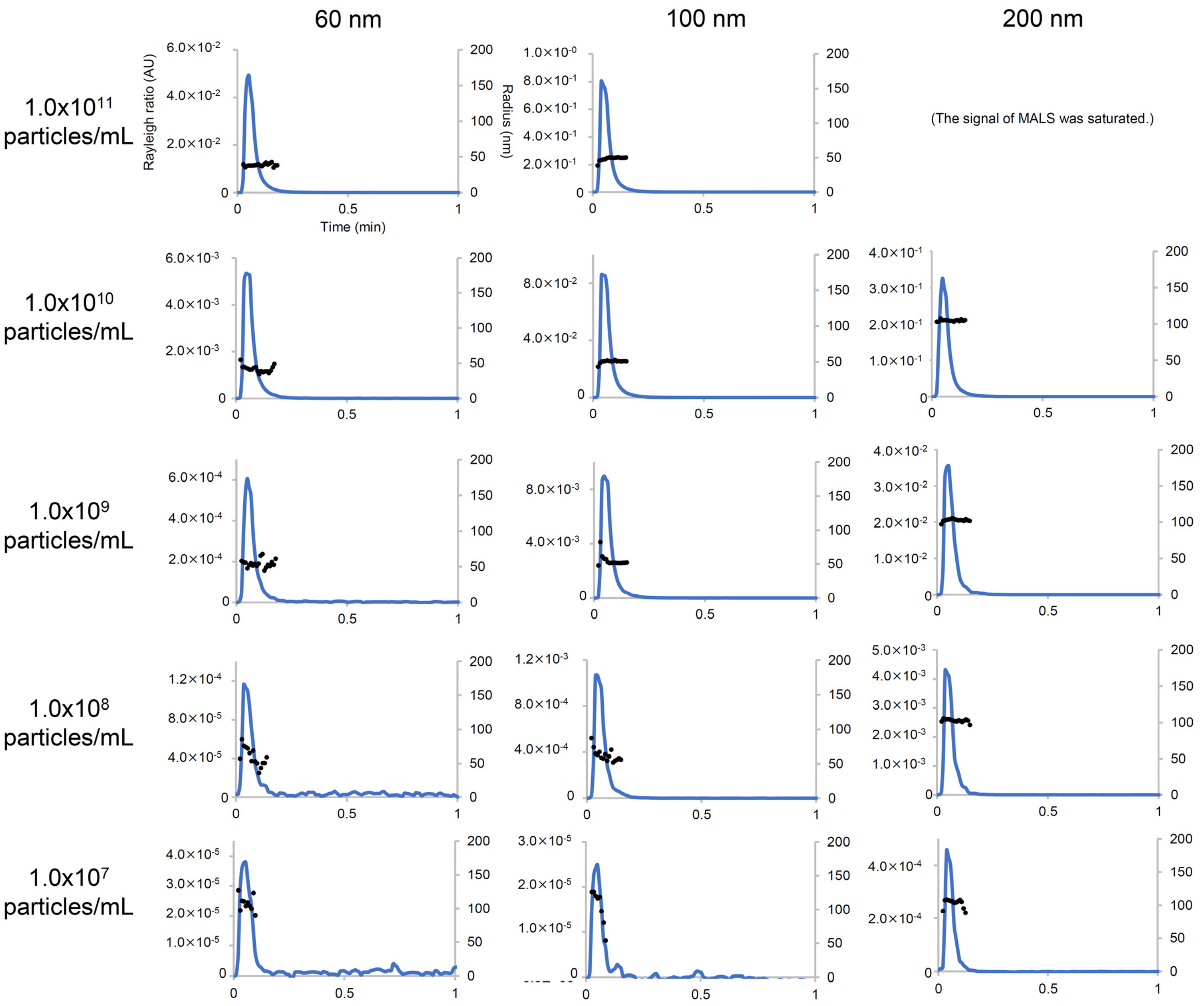

Supplementary Figure S4. Multi-angle light scattering (MALS) measurements of size standard particles. The particle sizes of the 60-, 100, and 200 nm size standard particles were measured by MALS. Ten μL of each sample injected into the HPLC system without the SEC column were analyzed by MALS. The measurements were repeated three times, and the mean diameter and RSD were used to evaluate the MALS performance on the particle size measurement.

Supplementary Table S1. Effect of dilution on particle size measurements of EV samples.

	Sample concentration (particles/mL)	UF			PS			SG		
		Diameter (nm)[a)]	RSD (%)		Diameter (nm)[a)]	RSD (%)		Diameter (nm)[a)]	RSD (%)	
NTA	5.00×10^{8}	101.3	2.8		90.7	7.7		110.1	0.9	
	2.50×10^{8}	96.3	4.2		90.9	8.3		107.6	2.2	
	1.25×10^{8}	97.2	3.6		92.7	7.0		104.0	0.9	
	6.25×10^{7}	94.0	6.3		94.1	9.6		107.1	7.6	
	3.13×10^{7}	98.5	2.2		97.4	5.2		103.9	3.3	
	Sample concentration (particles/mL)	UF			PS			SG		
		Diameter (nm)[a)]	RSD (%)	PDI	Diameter (nm)[a)]	RSD (%)	PDI	Diameter (nm)[a)]	RSD (%)	PDI
DLS	2.00×10^{9}	67.0	3.2	0.444	96.4	14.9	0.355	101.7	2.4	0.295
	1.00×10^{9}	70.7	3.9	0.450	96.5	21.1	0.375	104.4	10.4	0.319
	5.00×10^{8}	79.6	9.5	0.512	87.4	13.2	0.465	112.1	7.3	0.382
	2.50×10^{8}	86.8	16.1	0.590	71.7	23.3	0.611	124.0	24.6	0.422
	1.25×10^{8}	85.3	31.8	0.551		- [b)]		110.2	32.9	0.595
	Sample concentration (particles/mL)	UF			PS			SG		
		Diameter (nm)[a)]	RSD (%)		Diameter (nm)[a)]	RSD (%)		Diameter (nm)[a)]	RSD (%)	
SEC-MALS (Main peaks)	4.00×10^{9}	97.4	3.9		76.2	4.6		112.7	0.7	
	2.00×10^{9}	99.2	5.1		85.3	15.2		97.5	8.5	
	1.00×10^{9}	110.1	0.5		84.9	23.2		112.9	12.5	
	5.00×10^{8}	96.4	8.4		160.5	29.5		109.6	10.7	
	2.50×10^{8}	171.2	31.2			- [b)]		148.2	7.7	
	Sample Concentration (particles/mL)	UF: Pre			UF: Post			SG: Pre		
		Diameter (nm)[a)]	RSD (%)		Diameter (nm)[a)]	RSD (%)		Diameter (nm)[a)]	RSD (%)	
SEC-MALS (sub peaks)	4.00×10^{9}	196.4	4.0		80.9	4.6		196.7	1.0	
	2.00×10^{9}	200.4	1.5		84.2	13.6		182.5	2.3	
	1.00×10^{9}	219.2	3.2		91.4	16.6		199.2	7.1	
	5.00×10^{8}	196.3	1.8		86.3	7.9		205.4	12.4	
	2.50×10^{8}	300.0	21.8		206.7	23.9		225.6	4.9	

[a)] Each value represents the average of three measurements.
[b)] The intensity of the scattered light was inadequate for DLS and SEC-MALS measurements.
DLS, dynamic light scattering; NTA, nanoparticle tracing analysis; SEC-MALS, size exclusion high-performance chromatography with multi-angle light scattering; PS, phosphatidylserine sample; SG, size-exclusion gravity column sample; UF, ultrafiltration sample; RSD, relative standard deviation.
